# The *Staphylococcus aureus* Protein Sbi Acts as a Complement Inhibitor and Forms a Tripartite Complex with Host Complement Factor H and C3b

**DOI:** 10.1371/journal.ppat.1000250

**Published:** 2008-12-26

**Authors:** Katrin Haupt, Michael Reuter, Jean van den Elsen, Julia Burman, Steffi Hälbich, Julia Richter, Christine Skerka, Peter F. Zipfel

**Affiliations:** 1 Department of Infection Biology, Leibniz Institute for Natural Product Research and Infection Biology, Jena, Germany; 2 Department of Biology and Biochemistry, University of Bath, Claverton Down, Bath, United Kingdom; 3 Friedrich Schiller University, Jena, Germany; Dartmouth Medical School, United States of America

## Abstract

The Gram-positive bacterium *Staphylococcus aureus*, similar to other pathogens, binds human complement regulators Factor H and Factor H related protein 1 (FHR-1) from human serum. Here we identify the secreted protein Sbi (*Staphylococcus aureus* binder of IgG) as a ligand that interacts with Factor H by a—to our knowledge—new type of interaction. Factor H binds to Sbi in combination with C3b or C3d, and forms tripartite Sbi∶C3∶Factor H complexes. Apparently, the type of C3 influences the stability of the complex; surface plasmon resonance studies revealed a higher stability of C3d complexed to Sbi, as compared to C3b or C3. As part of this tripartite complex, Factor H is functionally active and displays complement regulatory activity. Sbi, by recruiting Factor H and C3b, acts as a potent complement inhibitor, and inhibits alternative pathway-mediated lyses of rabbit erythrocytes by human serum and sera of other species. Thus, Sbi is a multifunctional bacterial protein, which binds host complement components Factor H and C3 as well as IgG and β_2_-glycoprotein I and interferes with innate immune recognition.

## Author Summary

*Staphylococcus aureus* is a Gram-positive bacterium that can live as a commensal but can also cause severe life threatening infections in humans. Upon infection the bacterium is attacked by the host immune system, and in particular by the complement system which forms the immediate, first defence line of innate immunity. In order to survive, *S. aureus* has developed multiple evasion strategies and uses several virulence factors to evade and inactivate the host complement attack. Here we show that this pathogen binds the host complement regulators Factor H from human serum with the secreted and surface exposed Sbi protein, by a—to our knowledge—new type of interaction. Factor H binds to Sbi in combination with another host complement protein C3, C3b or C3d, and forms tripartite Sbi∶C3∶Factor H complexes. As part of this tripartite complex, Factor H is functionally active and inhibits further complement activation. Sbi, by recruiting Factor H and C3b, acts as a potent complement inhibitor, and inhibits alternative pathway-mediated lyses of rabbit erythrocytes by human serum and sera of different species. Thus, Sbi is a multifunctional bacterial protein, which binds host complement components Factor H and C3 as well as IgG and β_2_-glycoprotein I and interferes with innate immune recognition.

## Introduction

In order to establish an infection pathogens have developed multiple mechanisms to avoid immune recognition and to escape host immune attack [1],[2]. Complement, which mediates a powerful immediate innate immune defense of vertebrate hosts, is activated, within seconds upon entry of a foreign invader [1],[2]. Activation of the complement system occurs through three pathways, the alternative, the classical, or the lectin binding pathway. The activated system cleaves the central complement protein C3 into the fragments C3a and C3b, and deposits C3b onto the surface of a microbe, which normally results in opsonization and elimination of the microbe by phagocytosis. This surface deposited C3b initiates further activation of the complement cascade and results ultimately in the formation of the membrane attack complex (MAC), which forms a pore in the membrane and destroys the microbe by complement-mediated lyses. However for Gram positive bacteria MAC mediated lyses seems of minor significance. The cleavage products C3a and C5a serve as potent anaphylatoxins, which attract immune effector cells to the site of infection. Non-pathogenic microbes are effectively killed and eliminated by the complement system [3].

In order to restrict complement activation to the surface of an invading microbe host cells are protected from complement attack by membrane bound and soluble regulators. Factor H is the major fluid-phase complement regulator that controls alternative pathway activation at the level of C3. The 150-kDa Factor H protein is exclusively composed of 20 structural repetitive protein domains, termed short consensus repeats (SCR) [4]. Factor H is a member of a protein family, that includes the Factor H like protein 1 (FHL-1), encoded by an alternatively spliced transcript of the Factor H gene, and five Factor H related proteins (FHRs) that are encoded by separate genes [5]. Factor H controls complement activation by acting as a cofactor for the serine protease Factor I, which cleaves surface-bound C3b into iC3b. In addition, by competing with Factor B for C3b binding Factor H accelerates the decay of the alternative pathway C3 convertase. Thus, Factor H blocks C3b deposition and amplification of the complement cascade on the cell surface [5],[6].

In order to survive and to establish an infection, pathogens need to inhibit the host complement attack and apparently utilize diverse escape mechanisms. Several pathogens acquire host fluid-phase complement regulators, like Factor H, FHL-1, FHR-1 and C4b-binding protein (C4BP) from host plasma and body fluids. Bound to the surface of a pathogen, these host regulators retain complement regulatory functions, and inhibit complement activation. Therefore, acquisition of host regulators masks the pathogenic surface, which results in survival of the pathogen [7],[8].

This common strategy of complement evasion has been identified for multiple pathogens, including Gram-positive and Gram-negative bacteria, human pathogenic fungi, parasites and viruses and several of the corresponding surface proteins were identified [1]. The vast majority of these pathogenic surface proteins bind additional host plasma proteins and display multiple functions. The M protein of *Streptococcus pyogenes* binds the complement regulators Factor H, FHL-1 and C4BP as well as other plasma proteins, i.e. plasminogen, fibronectin, thrombin, fibrinogen, IgA, IgG and kininogen [1], [9]–[14]. The *Candida albicans* surface protein Glyceratphosphat-Mutase 1 (Gpm1) binds Factor H, FHL-1 and plasminogen [15]. In addition, Complement Regulator Acquiring Surface Protein 1 (CRASP-1) of *Borrelia hermsii* and Tuf of *P. aeruginosa*, bind Factor H, FHR-1 and plasminogen [16],[17]. The additional Factor H binding pathogenic surface proteins e.g. CRASP1 of *Borrelia burgdorferei*, PspC of *S. pneumoniae* and porin protein 1A of *Neisseria gonorrhoeae* are candidates for combined Factor H and plasminogen binding [18]–[20]. These pathogenic surface proteins display multiple functions and interfere with the complement regulation and coagulation. Thus, multiple or potentially all pathogens acquire soluble host factors and utilize these proteins for immune evasion [1].

*S. aureus* is a major human pathogen responsible for hospital- and community-acquired infection. The Gram-positive bacterium permanently colonizes the human skin and mucous membranes of approximately 20% of the population [21]. Once the pathogen has crossed host immune barriers *S. aureus* can cause superficial skin infection, toxin-mediated diseases or serious invasive infections depending on the interaction of the pathogen's virulence factors and the defense mechanisms of the host [22]. The pathogen utilizes complex strategies to survive and disseminate within the host and expresses several virulence factors to block both innate and adaptive immune response [23].

*S. aureus* utilizes several proteins to control and evade the host complement attack. The cell wall-anchored protein A (SpA) binds the Fc region of IgG [24]. *S. aureus* expresses the zymogen staphylokinase, that cleaves human plasminogen into active plasmin, which in turn cleaves IgG. In both cases recognition of the pathogen by C1q, the initial component of the classical complement activation pathway, is inhibited [25],[26]. Sbi is an additional staphylococcal IgG-binding protein that similar to SpA interacts with the Fc part of IgG [27]. Furthermore, Sbi binds β_2_-glycoprotein I, which is also termed apolipoprotein H [28]. Recently, additional effector molecules of *S. aureus* are identified, that directly interfere with complement activation at the level of C3. The extracellular fibrinogen-binding protein (Efb), the Efb homologous protein (Ehp), and the extracellular complement-binding protein (Ecb), bind C3 and C3d, prevent further activation of C3b and consequently block the activity of C3b-containing convertases [29],[30],[31],[32],[33]. The staphylococcal complement inhibitor (SCIN) acts on surface-bound C3 convertases, C3bBb and C4b2a, by stabilizing these complexes, thereby reducing the enzymatic activity [34],[35].

Here we show binding of Factor H and FHR-1 to the surface of intact *S. aureus* and in addition identify the secreted staphylococcal Sbi protein as a Factor H binding protein. Native Factor H from human serum binds to Sbi, and this binding is mediated by a second serum factor, which was identified as C3. Factor H binding is increased in the presence of C3b or C3d suggesting formation of a tripartite complex. This complex blocks activation of the alternative complement pathway. The Factor H binding site of Sbi which was located to domains III and IV is distinct from the IgG binding sites which are contained in the N-terminal domains I and II [28]. Here, we demonstrate a novel mechanism for Factor H binding by Sbi. Sbi forms a tripartite complex with Factor H and C3b or C3d and this complex interferes with complement activation.

## Results

### Factor H binds to the surface of *S. aureus*

**Figure 1:**
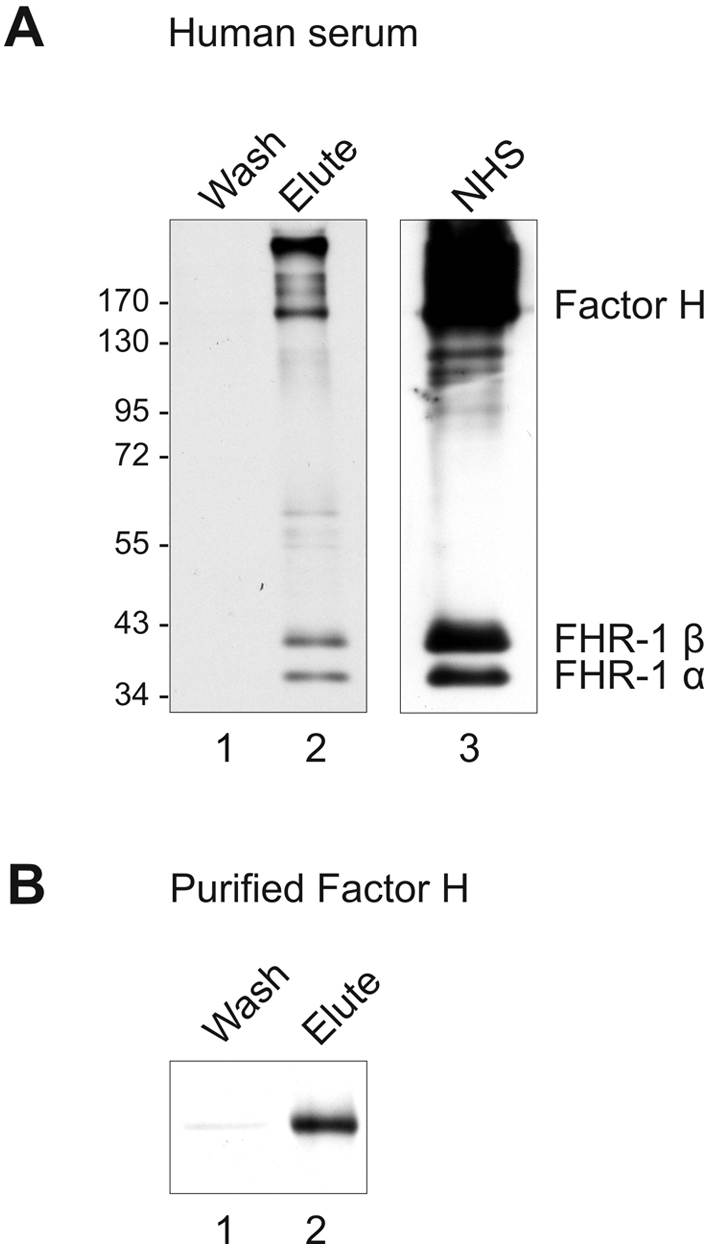
Adsorption of Factor H and FHR-1 to intact *S. aureus*. Cells of *S. aureus* strain H591 were incubated in human serum (A) or with purified Factor H (B). After extensive washing, bound proteins were eluted, separated by SDS-PAGE and analyzed by Western blotting using polyclonal Factor H antiserum. (A) In the eluted fraction (lane 2) polyclonal Factor H antiserum reacted with three bands of 150, 43 and 37 kDa representing Factor H, FHR-1β and FHR-1α, respectively. The same proteins were also identified in human serum (lane 3). (B) Purified Factor H bound to the bacteria and was detected in the elute fraction (lane 2). The mobility of the marker proteins is indicated. NHS, normal human serum.

In order to analyze binding of host complement regulators to *S. aureus*, strain H591 was incubated in human serum. After extensive washing bound proteins were eluted, separated by SDS-PAGE, transferred to a membrane and analyzed by Western blotting. This approach identified three bands of 150, 43 and 37 kDa, which represent Factor H, FHR-1β and FHR-1α, respectively (Figure 1A, lane 2). These proteins were absent in the final wash fraction, thus suggesting specific binding (Figure 1A, lane 1 and lane 2). The same proteins were also identified in human serum (Figure 1A, lane 3). When bacteria were incubated with purified Factor H binding of the purified protein was also detected in the eluted fraction (Figure 1B, lane 2).

### Factor H binds to secreted protein Sbi

**Figure 2:**
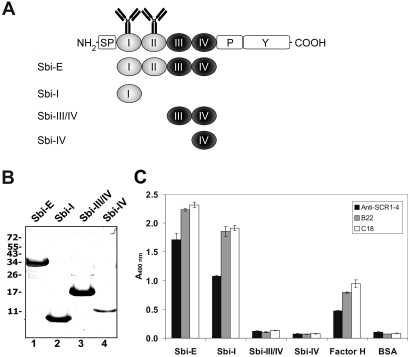
Sbi constructs and antibody binding to Sbi. (A) Schematic structure of Sbi and of Sbi deletion constructs used in the experiments. The IgG binding domains Sbi-I and Sbi-II are shown in white and the non IgG binding, but Factor H, C3 binding domains (domains III and IV) are shown in black. In addition the position of the signal peptide (SP), the prolin-rich (P) and the tyrosine-rich (Y) regions are indicated. (B) Sbi deletion constructs expressed in *E. coli* were purified by nickel chromatography, separated by SDS PAGE and identified by silver staining. Based on mobility of the marker proteins the molecular mass of the fragments is as follows. Sbi-E 34 kDa; Sbi-I 9.7 kDa; Sbi-III/IV 17 kDa and Sbi-IV 11 kDa. (C) The IgG binding fragments Sbi-E and Sbi-I mediate unspecific binding of the Factor H∶IgG complex. O binding of purified Factor H is detected to the Non-IgG binding domains Sbi-III/IV and Sbi-IV. Factor H was immobilized and used a positive control. Binding of Factor H to immobilized recombinant constructs Sbi-E, Sbi-I, Sbi-III/IV and Sbi-IV was identified with polyclonal Factor H antiserum (anti-SCR 1–4) and specific mABs B22 and C18 by ELISA.

In order to characterize the bacterial ligand mediating this interaction we hypothesized that the staphylococcal Sbi protein might represent the binding protein. The N-terminal region of Sbi (i.e. Sbi-E) is composed of four domains and includes the IgG binding domains I and II, whereas domains III and IV lack antibody binding properties (Figure 2A and C) ([28], Burman et al. JBC in press). IgG binding of Sbi-E and Sbi-I was confirmed for one polyclonal antiserum and two monoclonal antibodies (mABs), which are directed to Factor H (Figure 2C, columns 1 and 2). Antibody binding was rather strong and exceeded the reactivity for the specific ligand Factor H (Figure 2, compare columns 5 and 1).

### Factor H binding and localization of the Factor H binding domains in Sbi

**Figure 3:**
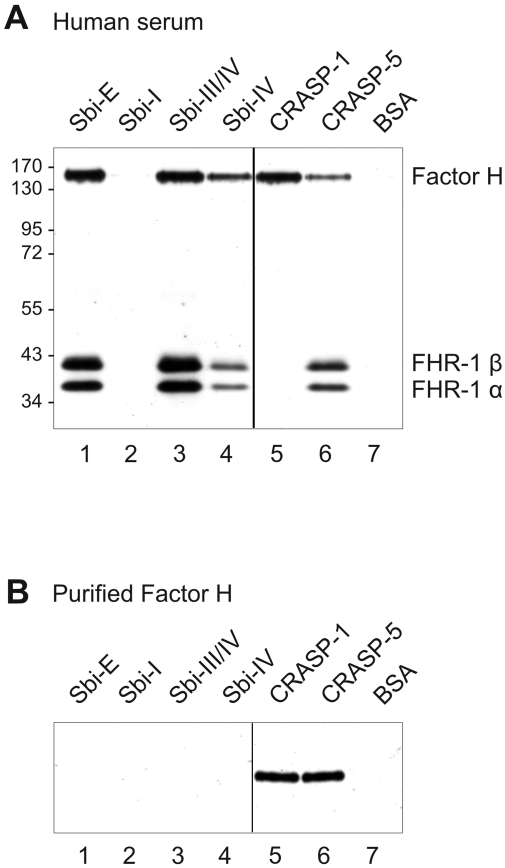
Binding of Factor H and FHR-1 to Sbi. (A) CEWA was used to analyze interaction of host complement regulators with the IgG binding Sbi protein. Sbi-E, Sbi-I, Sbi-III/IV and Sbi-IV were immobilized onto the surface of a microtiter plate and human serum was applied. After extensive washing bound proteins were eluted, separated by SDS-PAGE and identified by Western blotting based on their mobility and their reactivity with mAB C18 that is specific for the C-terminal SCR domain of Factor H and FHR-1. The borrelial Factor H binding CRASP-1, the Factor H/FHR-1 binding CRASP-5 and BSA were used as controls. The mobility of the marker proteins is indicated. (B) Purified Factor H was used in the same assay.

Sbi is an IgG binding protein, therefore Sbi-E and Sbi-I interaction with additional ligands cannot be studied by standard ELISA. Consequently we used the previously described combined ELISA and Western blot approach (CEWA) to study binding of human serum proteins to Sbi [36]. CEWA, which allows the identification of Sbi bound serum proteins by size and by reactivity with specific antisera, revealed that Factor H as well as both FHR-1α and FHR-1β bind to Sbi-E, comprising domains I–IV (Figure 3A, lane 1). Both Factor H and FHR-1α/FHR-1β bound to the deletion constructs Sbi-III/IV and with lower intensity to Sbi-IV (Figure 3A, lanes 3 and 4). The IgG binding domain Sbi-I did not bind the host complement regulators (Figure 3A, lane 2). As described previously Factor H bound to borrelial CRASP-1 and CRASP-5 and FHR-1α/FHR-1β to CRASP-5 (Figure 3A, lanes 5 and 6) [36].

Having demonstrated binding of Factor H, FHR-1α and FHR-1β from human serum to Sbi via domains III and IV, we wanted to confirm this interaction with purified proteins. However purified Factor H did not bind to Sbi, but did bind to CRASP-1 and CRASP-5 (Figure 3B). These results suggest that binding of Factor H to Sbi is mediated by an additional serum factor.

### Identification of a serum factor that mediates Factor H∶Sbi interaction

**Figure 4:**
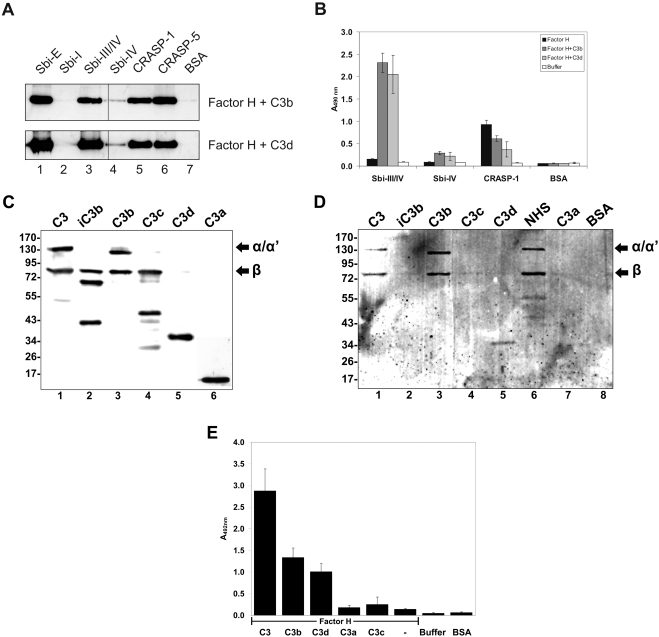
Binding of Factor H and C3 to Sbi. (A) Sbi-E, Sbi-I, Sbi-III/IV and Sbi-IV, CRASP-1 and CRASP-5 of *Borrelia burgdorferi* and BSA were immobilized and incubated with Factor H together with C3b or C3d. After washing bound proteins were eluted from the wells, separated by SDS-PAGE and analyzed by Western blotting using the mAB C18. In the presence of C3b or C3d Factor H bound to Sbi-E, Sbi-III/IV, Sbi-IV, CRASP-1 and to CRASP-5. (B) Sbi-III/IV, Sbi-IV, CRASP-1 and BSA were immobilized and binding of Factor H alone, Factor H and C3b, or Factor H and C3d was assayed by ELISA using polyclonal Factor H antiserum. Again in the presence of C3b or C3d Factor H bound to Sbi-III/IV, to Sbi-IV weakly and to CRASP-1. Factor H alone did bind to CRASP-1. (C) Western blot showing C3 and its degradation products which were used for the CEWA approach. (D) Binding of Sbi to C3 and various degradation. Sbi-E was immobilized and the indicated C3 forms were assayed for binding in the presence of Factor H. Afterward protein were eluted, separated by SDS-Page, transferred to a membrane and C3 fragments were visualized with specific antiserum. C3, C3b and C3d bound to Sbi-E (lanes 1, 3 and 5); and iC3b, C3c and C3a did not bind (lanes 2, 4 and 7). In addition NHS derived C3 bound to Sbi-E (lane 6). BSA was used as negative control (lane 8). (E) Sbi-III/IV was immobilized and binding of Factor H in combination with either C3, C3b, C3d, C3a and C3c, or Factor H alone was measured using polyclonal Factor H antiserum.

In order to identify the additional serum factor that mediates binding of the host complement regulatory, we hypothesized that the central complement component C3, which binds to the staphylococcal inhibitors Efb, Ehp and Ecb [29],[30],[32] might be such a mediator. Consequently binding of purified Factor H in the presence of the complement proteins C3b and C3d was analyzed by CEWA. When coincubated with either C3b or C3d Factor H bound to Sbi-E, Sbi-III/IV and Sbi-IV, but not to Sbi-I (Figure 4A). This binding suggests that Sbi forms a tripartite complex with Factor H and C3.

Factor H binds to domains III and IV of Sbi, but not to the IgG binding domain I. The interaction to the non-IgG binding domains was confirmed by standard ELISA. Purified Factor H together with C3b or C3d bound to Sbi-III/IV (Figure 4B, columns 1). Binding of Factor H together with C3b or C3d to Sbi-IV was rather low. In this assay the binding of Factor H together with C3b/C3d to Sbi-III/IV was more pronounced as compared to borrelial CRASP-1 (Figure 4B, compare columns 1 and 3). In addition the C3 fragment responsible for complex formation with Factor H was assayed by CEWA and ELISA (Figure 4D and 4E). The C3d-containing fragments C3, C3b and C3d mediate complex formation of Factor H with Sbi, but not C3a, C3c nor to iC3b. This result reveals a novel mechanism of capturing host immune regulators, as Sbi binds Factor H in combination with a second host ligand, namley C3.

### Characterization of the Sbi∶C3∶Factor H tripartite complex

**Figure 5:**
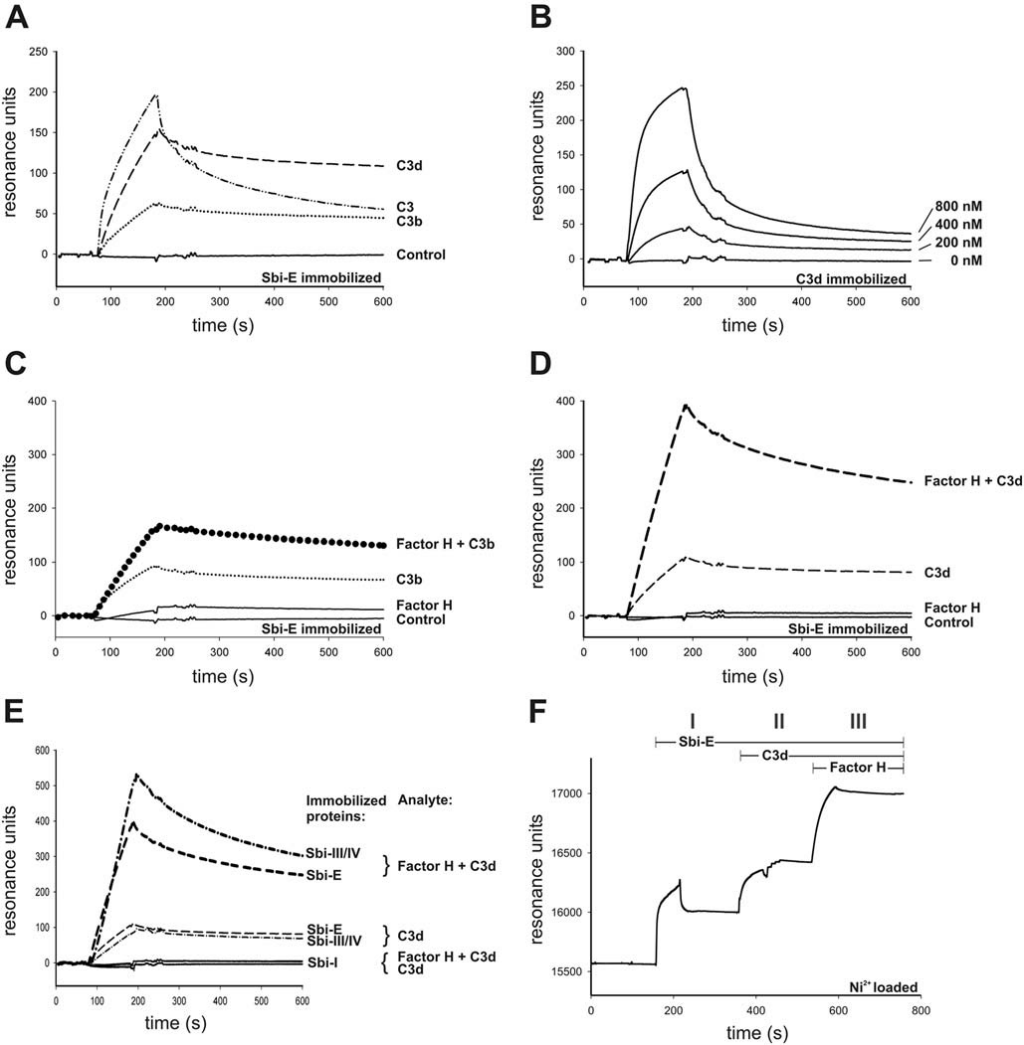
Analyzing Sbi∶C3 interaction by surface plasmon resonance. (A) Sbi-E was immobilized and C3, C3b and C3d were applied in fluid phase. C3 showed strong association and after removal of the analyte a relative fast dissociation (dash-dotted line). C3b showed a slower association profile as compared to unprocessed C3, however the complex was rather stable (dotted line). In addition C3d showed a prominent association profile and the complex was rather stable (dashed line). (B) Dose-dependent interaction of Sbi-E to immobilized C3d. (C) Effect of C3b on Factor H-Sbi interaction. Sbi-E was immobilized and binding of the single components Factor H or C3b, or of a combination of the two proteins Factor H and C3b was analyzed. (D) Effect of C3d on Factor H-Sbi interaction. Sbi-E was immobilized and binding of the single components Factor H or C3d, or of a combination of the two proteins Factor H and C3d was analyzed. As identical concentrations were used the binding profiles can be directly compared and thus demonstrate stronger interaction of the complex in the presence of C3d as compared to C3b. (E) The various fragments Sbi-E, Sbi-I and Sbi-III/IV were coupled to the surface of a sensor chip and binding of proteins Factor H alone or C3d alone or in combination was analyzed. C3d bound to Sbi-E and Sbi-III/IV with comparable intensities, but did not bind to Sbi-I. The Factor H∶C3d complex showed stronger binding to Sbi-E and Sbi-III/IV, but did not bind to Sbi-I. (F) Subsequent association of Sbi-E, C3d and Factor H to an NTA-chip forming a stable Sbi-E∶C3d∶Factor H complex.

Having identified staphylococcal Sbi as a protein that binds the host complement components Factor H together with C3b or C3d, we analyzed C3 binding and tripartite complex formation in more detail. First binding of the various forms of C3 was analyzed to immobilized Sbi-E in real time using surface plasmon resonance. C3 showed a strong association and a relative fast dissociation (Figure 5A: C3). C3b, used at the same molar ratio showed slower association, but the Sbi∶C3b complex was rather stable (Figure 5A: C3b). In addition C3d, the degradation product of C3, showed a more pronounced association and also a slow rate of dissociation (Figure 5A: C3d). This slow dissociation profile of both C3d and C3b suggests a high stability of the Sbi∶C3b and Sbi∶C3d complexes.

Based on the apparent stronger association of C3d to Sbi-E, this interaction was analyzed in more detail. Sbi-E showed a dose-dependent binding to immobilized C3d when used at a range of 200, 400 and 800 nM (Figure 5B). The same dose-dependent binding was observed in a reverse setting with immobilized Sbi-E (data not shown). These results demonstrate that C3, C3b and C3d bind directly to the staphylococcal Sbi.

In order to further analyze the interaction and complex formation Sbi-E representing domains I-IV were immobilized and complex formation was followed in real time. In this setting purified Factor H bound rather weakly to immobilized Sbi-E, while C3b binding was stronger (Figure 5C). An increase was observed in the presence of both Factor H and C3b confirming formation of a tripartite complex (Figure 5C). Formation of the tripartite complex was also analyzed with Factor H and C3d (Figure 5D). In this setting binding of C3d was similar to that of C3b (compare Figure 5D and Figure 5C) and based on the RLUs the tripartite Sbi∶C3d∶Factor H complex showed more pronounced interaction.

Binding and tripartite complex formation was analyzed to immobilized Sbi-constructs, i.e. Sbi-E, Sbi-I and Sbi-III/IV, to localize the C3 binding domains in Sbi. C3d did not bind to the IgG binding domain Sbi-I, but to Sbi-E and also to the construct Sbi-III/IV (Figure 5E). C3d interaction to Sbi-E and Sbi-III/IV was comparable, thus confirming the role of domains III and IV for the contact. Based on the strong interaction of the Sbi∶C3d∶Factor H complex to Sbi-E and to Sbi-III/IV (Figure 5E) it is concluded that the C3/Factor H interaction region of Sbi is located exclusively in Sbi domains III and IV.

To characterize the formation of Sbi∶C3d∶Factor H complex Sbi-E was coupled to an NTA-chip and complex formation was followed upon sequential addition of C3d and Factor H. Immobilization of Sbi-E was observed (Figure 5F, phase I) and upon addition of C3d formation of the Sbi∶C3d complex was followed in real time (Figure 5F, phase II). Upon addition of Factor H, a further association was detected by the increase in the surface plasmon resonance signals. These results demonstrate that Factor H binds directly to the Sbi∶C3d complex and that Factor H does not compete with C3b for Sbi-E binding (Figure 5F, phase III). The observed mass increase at the surface of the sensor chip during association of Factor H to the Sbi∶C3d complex was higher than that of Factor H to immobilized C3d (data not shown).

### Localization of the Sbi binding domains within Factor H

**Figure 6:**
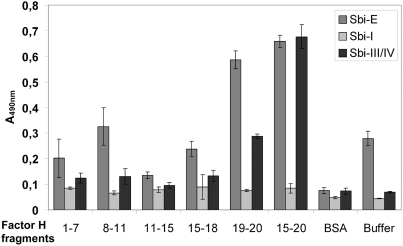
Localization of the Sbi binding regions within Factor H. The indicated Factor H deletion constructs were immobilized to the surface of a microtiter plate and the Sbi deletion constructs together with C3b were added. Binding was assayed by ELISA using polyclonal Factor H antiserum. SCRs 15–20 and SCRs 19–20 bound to Sbi-E and Sbi-III/IV but not to Sbi-I. The additional Factor H deletion mutants did not bind to Sbi.

To further characterize this novel type of Factor H acquisition with C3, we decided to identify the Factor H domains that are involved in this interaction. Factor H deletion constructs were immobilized and used in an ELISA experiment. In the presence of C3b, Sbi-E and Sbi-III/IV, but not to Sbi-I bound to immobilized Factor H SCRs 19–20 and SCRs 15–20 (Figure 6, columns 5 and 6). In addition Sbi-I did not bind to any Factor H deletion construct. Thus the Sbi binding site was localized within the C-terminal surface binding region of Factor H, within SCRs 19–20 and is restricted to Sbi domains three and four.

### Factor H fixed in the Sbi∶C3b tripartite complex displays cofactor activity

**Figure 7:**
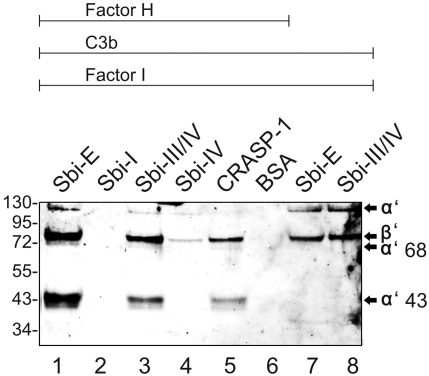
Factor H retains cofactor activity within the Sbi∶C3b∶Factor H complex. Sbi-E, Sbi-I, Sbi-III/IV and Sbi-IV, and the borrelial CRASP-1 were immobilized to the surface of a microtiter plate. Factor H and C3b were added and following extensive washing Factor I was applied. After 30 min incubation the mixture was harvested and separated by SDS-PAGE. C3b degradation was analyzed by Western blotting using a polyclonal C3 antiserum. The mobility of the α' and β' chain of C3b and the cleavage products α'68 and α'43 are indicated. Factor H mediated cofactor activity is detected when the complex is coupled to Sbi-E and Sbi-III/IV (lanes 1 and 3). The borrelial CRASP-1 protein and BSA were used as controls (lane 5 and 6). In the absence of Factor H C3b remains intact (lanes 7 and 8).

Factor H bound to pathogenic ligands maintains complement regulatory activity which relates to complement evasion [1]. It was therefore of importance to assay if Factor H fixed in this tripartite complex is functionally active and has complement regulatory activity. Factor H and C3b were incubated simultaneously with immobilized Sbi-E or the deletion fragments Sbi-I, Sbi-III/IV and Sbi-IV. Subsequently, Factor I was added and the mixture was incubated further. Following this treatment the proteins were eluted, separated by SDS-PAGE and after transfer to a membrane the C3b degradation products were identified by Western blotting. Factor H bound to Sbi-E in the presence of C3b displayed cofactor activity as indicated by the disappearance of the α' band and the appearance of the α'68- and α'43 bands (Figure 7, lane 1). The same degradation profile of C3b was observed when Factor H was bound to Sbi-III/IV (Figure 7, lane 3) or to borrelial CRASP-1 (Figure 7, lane 5). In the absence of Factor H no degradation of C3b was observed (Figure 7, lanes 7 and 8). These results show that Factor H attached to Sbi in a tripartite complex maintains complement regulatory activity.

### Characterization of the binding mechanism within the tripartite complex

**Figure 8:**
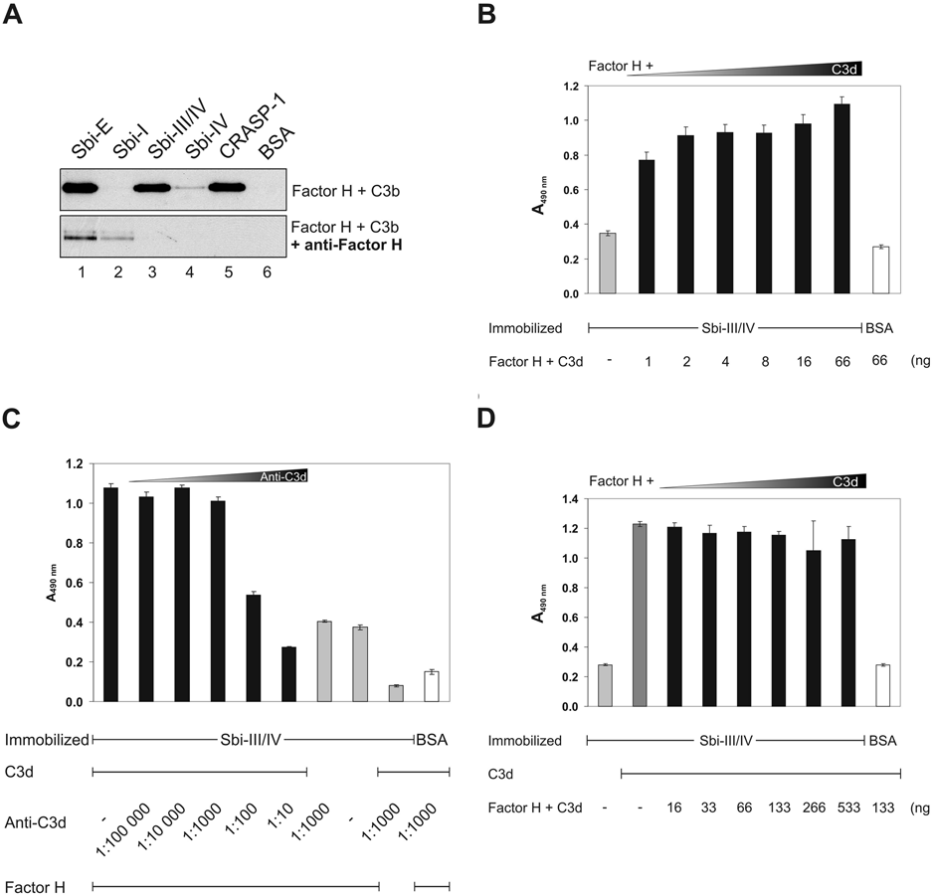
Characterization of the tripartite Sbi∶Factor H∶C3 complex. (A) Polyclonal Factor H antiserum blocks the formation of the Sbi∶C3b∶Factor H complex. Sbi-E, Sbi-I, Sbi-III/IV and Sbi-IV were immobilized onto the surface of a microtiter plate and Factor H and C3b (upper panel) or Factor H, C3b preincubated with polyclonal Factor H antiserum (lower panel) were added. After extensive washing the bound proteins were eluted, separated by SDS-PAGE and Factor H was identified by Western blotting using polyclonal SCR 1–4 antiserum. Polyclonal Factor H antiserum inhibited formation of the tripartite complex. CRASP-1 of *B. burgdorferi* and BSA were used as controls. (B) Formation of the tripartite Sbi-III/IV∶C3d∶Factor H complex was studied by ELISA. In order to determine the minimal amount of C3d required for increased Factor H binding Sbi-III/IV was immobilized and Factor H in combination with increasing amounts of C3d were added. Binding of Factor H was recorded. (C) Sbi-III/IV and BSA were immobilized, incubated with C3d and increasing dilutions of C3d antiserum was added. After washing Factor H was added and binding was assayed. (D) Saturating concentrations of C3d (0.2 µg) were added to immobilized Sbi-III/IV and BSA. Simultaneously Factor H was preincubated with increasing amounts of C3d and the protein mixture was added to the immobilized Sbi-III/IV∶C3d complexes. Subsequently, Factor H binding was measured.

Tripartite Sbi∶C3d∶Factor H complexes represent –to our knowledge- a novel mechanism for Factor H attachment. Factor H has a C3b/C3d binding region within the C-terminal recognition region, which also forms the major contact with Sbi. Therefore we asked whether the tripartite complex is based on a sandwich type interaction, by which Sbi binds first intact C3, C3b or C3d and then Factor H. Alternatively a tripartite complex may be formed, in which Factor H directly contacts Sbi and C3. Inhibition experiments were performed to test this hypothesis and to characterize this interaction in more detail. First Factor H and C3b were incubated in the presence of Factor H antiserum and Factor H binding to immobilized Sbi was studied. Preincubation of Factor H with the specific antiserum decreased binding to Sbi-E and blocked binding to the fragments Sbi-III/IV and Sbi-IV (Figure 8A, lower panel). The weak binding of antiserum treated Factor H to intact Sbi-E and to Sbi-I is explained by binding of the Factor H∶IgG complex via the IgG binding site of Sbi located within domain I.

First binding of Factor H to immobilized Sbi-III/IV in the presence of increasing amounts of C3d was studied. Already 1 ng of C3d, resulting in a molar Factor H∶C3d ratio of 25∶1 enhanced Factor H∶Sbi interaction (Figure 8B).

Secondly, Sbi-III/IV was immobilized, C3d was added and Sbi-III/IV bound C3d was blocked with increasing amounts of specific C3d antiserum. Subsequently, the binding of Factor H was analyzed. Factor H binding was not impaired with antisera titers up to 1∶1000, and was reduced but not completely blocked at the highest titers (1∶100 and 1∶10) (Figure 8C). This result shows direct binding of Factor H to Sbi and indicates that the presence of C3d, Sbi enhances formation of the tripartite complex.

Similarly, Sbi-III/IV was immobilized and a saturating amount of C3d was bound. In order to block C3d binding sites on the Factor H protein, Factor H was preincubated with increasing concentrations of C3d prior to binding. The preincubated Factor H∶C3d complexes were added to the immobilized Sbi∶C3d complexes and after incubation Factor H binding was analyzed. Again tripartite Sbi∶C3d∶Factor H complexes were detected and complex formation was independent of the amount of C3d used for preincubation (Figure 8D). This result is in agreement with a direct Factor H∶Sbi contact. In summary the inhibition and blocking experiments reveal that Factor H binds directly to Sbi and that binding is assisted by C3d.

### Sbi is a complement inhibitor

**Figure 9:**
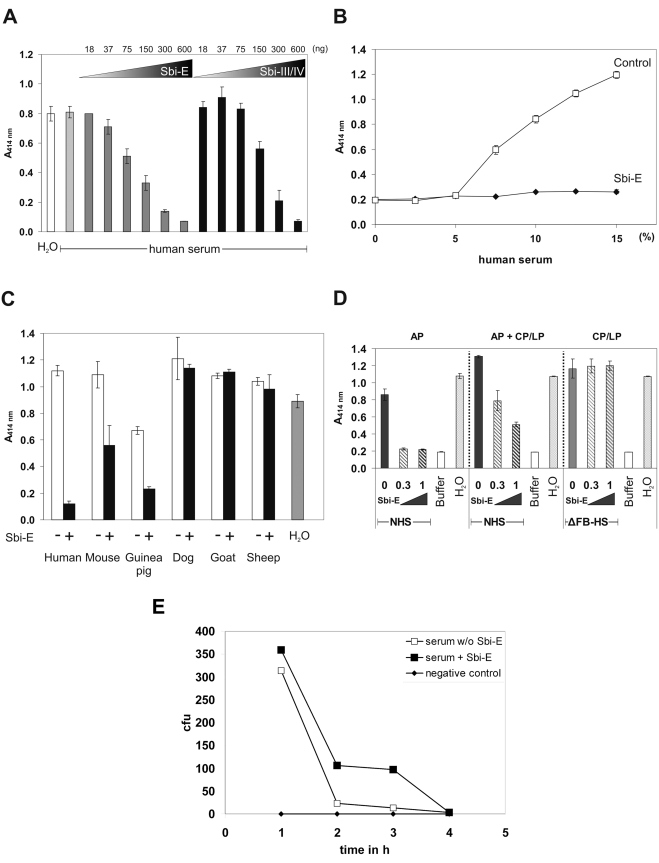
Sbi is a complement inhibitor. (A) Alternative pathway-mediated hemolysis of rabbit erythrocytes was assayed in the presence of increasing amounts of Sbi-E (columns 3–8) or Sbi-III/IV (columns 9–14). Hemolysis of rabbit erythrocytes with ddH_2_O is shown in column 1; hemolysis in human serum is shown in column 2. (B) Hemolysis of rabbit erythrocytes following incubation in increasing concentrations of complement active human serum in the absence (open squares) or the presence of Sbi-E (filled rhombi). (C) Lysis of rabbit erythrocytes in serum (30%) derived from the indicated species; i.e. human, mouse, guinea pig, dog, goat and sheep. (D) Comparing the effect of Sbi on alternative pathway (AP)−, alternative, classical and lectin pathway (AP+CP/LP)−, as well as classical and lectin pathway (CP/LP)-mediated hemolysis of human serum on rabbit erythrocytes. Background lysis was subtracted for (A) and (C). NHS, normal human serum, ΔFB-HS, Factor B depleted human serum. (E) Sbi inhibits opsonophagocytosis of *S. aureus* by THP-1 macrophage. Bacteria were incubated in 40% NHS in the presence or absence of Sbi for 20 min. Bacteria were added to activated human THP1 macrophages and at the indicated times bacteria were recovered and the number of live bacteria was quantitated. bacteria were plated on agar and the colony formation was determined (CFU).

Staphylococcal Sbi forms a tripartite complex with host complement proteins Factor H and C3. Consequently the complement inhibitory activity of Sbi was assayed in a standard hemolysis assay, using human serum and rabbit erythrocytes. In this assay Sbi-E and also Sbi-III/IV inhibited complement-mediated lyses of rabbit erythrocytes in a dose-dependent manner. Complete inhibition was observed at a concentration of 600 ng of either Sbi-E or Sbi-III/IV (Figure 9A). In contrast, Sbi-I had no effect (data nor shown) indicating that C3b and Factor H binding is relevant for complement inhibitory activity. These results demonstrate that Sbi acts as a potent complement inhibitor. Hemolysis of rabbit erythrocytes in human serum was dose-dependent over a range from 5 to 15% and Sbi blocked hemolysis efficiently at all serum concentrations (Figure 9B).

To analyze the species range of Sbi-E the inhibitory effect of Sbi-E was tested using sera of different species. Complement mediated inhibition was observed in human, mouse and guinea pig sera, and no effect was detected in dog, goat and sheep sera (Figure 9C). Thus Sbi acts in human serum but also displays a broader species range.

Sbi is a potent complement inhibitor. Therefore we investigated the inhibitory effect of Sbi-E in all three complement pathways. Sbi-E clearly inhibited alternative pathway activity (Figure 9D, column 2 and 3). When all pathways were activated hemolysis was reduced in a dose-dependent manner, indicating that the alternative pathway, which was blocked by Sbi-E, is involved and that the classical and lectin pathway are unaffected (Figure 9D, columns 7 and 8). This effect was confirmed upon analyzing the impact on the classical and the lectin pathway. Sbi-E did not inhibit hemolysis of rabbit erythrocytes when complement was activated via the classical and the lectin pathway (Figure 9D, columns 12 and 13).

As Sbi inhibits complement we asked whether Sbi protects *S. aureus* from phagocytosis mediated killing. *S. aureus* was incubated with complement active human serum in presence or absence of Sbi-E. Subsequently bacteria were harvested and incubated together with activated phagocytic THP-1 macrophages. At the indicated times points bacteria were recovered and the survival rate was quantitated. The presence of Sbi increased bacterial survival (Figure 9E), thus indicating that the inhibitor Sbi protects bacteria from opsonisation and phagocytosis.

These results demonstrate that Sbi-E efficiently inhibits the alternative, complement pathway and aids in bacterial resistance against complement mediated phagocytosis.

## Discussion

The Gram-positive bacterium *S. aureus*, similar to other human pathogens binds the complement regulators Factor H and FHR-1 from human serum. We identify the staphylococcal Sbi protein as a ligand for the two host complement regulators. Apparently Sbi binds Factor H by a new mechanism, as this human regulator binds to Sbi together with C3, which likely results in formation of a tripartite Sbi∶C3∶Factor H complex. Arranged in this tripartite complex Factor H is functionally active and displays complement regulatory activity. Thus Sbi is a potent complement inhibitor, and inhibits the hemolytic activity of human and rodent serum on rabbit erythrocytes via the alternative pathway. Thus the multifunctional bacterial Sbi protein interferes with innate immune recognition, by acquisition of multiple host proteins in form of the complement components Factor H, C3 as well as IgG and β_2_-glycoprotein I.

Purified Factor H bound to intact bacteria, but dependent on the assay showed weak or even no binding to Sbi (compare Figure 1, Figure 3B and Figure 5C,D). This difference in binding suggests that intact *S. aureus* bacteria express an additional Factor H binding surface protein. The identification of this protein is subject to further studies. The staphylococcal Sbi protein was identified as a ligand for Factor H. However Factor H binding is enhanced in the presence of an additional complement protein C3. A tripartite Sbi∶C3b∶Factor H complex is formed (Figure 4 and Figure 5).

The Factor H contact region for Sbi is located within SCRs 19–20 (Figure 6). Very similar contact domains were identified for other microbial Factor H binding proteins, e.g. CRASP-1 and CRASP-2 of *B. burgdorferi*, Tuf of *P. aeruginosa* and Gpm1 of *C. albicans* [12],[14],[16],[37]. Inhibition experiments showed that polyclonal Factor H antiserum blocks Factor H binding to Sbi (Figure 8A). In the proposed tripartite complex the regulatory region of Factor H (i.e. SCRs 1–4) is freely accessible as demonstrated by the Factor I mediated cleavage of C3b (Figure 7).

The staphylococcal Sbi protein is composed of four globular N-terminal domains connected to a tyrosine-rich C-terminal domain via a prolin-rich region (Figure 2A) [38]. A recombinant fragment with domains I–IV (Sbi-E), as well as constructs Sbi-III/IV and Sbi-IV, but not Sbi-I bound Factor H in combination with C3b or C3d (Figure 4 and Figure 5), thus localizing the Factor H binding region to Sbi domains III and IV. As the Factor H/C3b binding sites in domain III and IV and the IgG binding sites in domain I and II are separated, the Sbi protein may simultaneously bind several host proteins.

The binding properties of Sbi are unique, as –to our knowledge– Sbi is the first bacterial protein identified that forms such a tripartite complex with Factor H and C3, C3b or C3d. It will be of interest to demonstrate whether other proteins of pathogen origin or virulence factors form similar tripartite complexes. The Sbi∶C3 interaction appears rather complex, as intact C3 and the two processed forms C3b and C3d display different binding profiles resulting in different stabilities (Figure 5A). C3d complexed to Sbi showed the highest binding intensity of binding, and C3b or C3 a lower interaction. In addition the rate constants of C3d and Sbi-III/IV when assayed by surface plasmon resonance did not fit a 1∶1 langmuir model of interaction, but rather fit a bivalent analyte model (Figure S2A and S2B, Figure S3).The proposed bivalent analyte interaction together with the different binding profiles for the three C3 forms suggest that C3 undergoes a conformational change upon binding to Sbi and exposes additional binding epitopes, which affect Sbi interaction, or that these C3b/C3d binding region(s) is/are differently accessible to the bacterial Sbi protein.

During complement activation C3 is cleaved, the C3 cleavage products bind to Sbi, increase Factor H binding and enhance the stability of the tripartite complex. Such a feed back regulation may increase the amount of inhibitory host regulators like Factor H at the site of infection and result in protection of the pathogen from complement attack and thus improves bacterial survival (Figure 9E).

This inhibition of the alternative pathway by Sbi indicates that Factor H bound to Sbi affects the C3 convertase. Within the tripartite complex Factor H displays complement regulatory activity (Figure 7) and seems responsible for hemolytic activity (Figure 9A and data not shown). This explains why Sbi domains III and IV display an inhibitory effect. Compared to the other staphylococcal complement regulators Efb and Ecb, Sbi does not interfere with the activity of the classical pathway and did not affect hemolysis mediated by the classical pathway (Figure 9D) [29]. Thus Sbi forms a tripartite complex with the two human complement proteins Factor H and C3, revealing- to our knowledge- a novel mechanism for complement inhibition.

The inhibitory activity of Sbi is not restricted to human complement as the protein also blocks complement of other species i.e. mouse and guinea pig. Demonstrating that Sbi is a staphylococcal virulence factor with a broader species range as compared to the human specific inhibitor SCIN, which acts specifically in the human system [29],[34].

Sbi is a potent complement inhibitor, which interferes with the hemolytic activity of human serum. In hemolytic assays with rabbit erythrocytes Sbi used at 2 µg/ml ( = 0.3 µg) exclusively blocked the alternative pathway whereas the classical and the lectin pathways were unaffected (Figure 9D). However when used at higher concentrations of 1000 µg/ml in an ELISA approach the Sbi-III/IV fragment blocks all three activation pathways of human complement but the Sbi-IV fragment is a specific inhibitor for the alternative pathway (Burman et al. JBC in press). This activity differs from that of SCIN and its homologues SCIN-B and SCIN-C, which affects all three complement pathways [29],[34].

The staphylococcal Sbi protein is a multifunctional protein which binds the complement effectors Factor H, FHR-1 and C3 and also the processed forms C3b and C3d, as well as IgG and β_2_-glycoprotein I. Thus Sbi mediates innate and adaptive immune escape (i) by acquiring host complement inhibitors, which correlates with the activation state of complement, (ii) by inhibiting complement activation at the level of alternative pathway C3 convertase, (iii) by binding and inactivation of IgG to avoid recognition by phagocytes, and (iv) most likely by blocking C3dg binding to complement receptor 2 (CR2) (Burman et al. JBC in press).

## Materials and Methods

### Bacterial strain and culture condition

*S. aureus* strain H591 (MSSA clinical isolate, UK) was grown at 37°C in tryptic soy broth (TSB, Sigma). The strain was characterized for the presence of Sbi on DNA and protein level (Figure S1A, S1B and S1C).

### Adsorption experiments

Overnight cultures of *S. aureus* were diluted to OD_600_ = 0.2 in TSB and incubated for about 1.5 h at 37°C to OD_600_ = 1.0 (approximately 1.2×10^9^ cfu). Cells (2×10^9^ cfu) were harvested by centrifugation (6000 g, 8 min at room temperature), resuspended in veronal buffered saline (GVB^2+^, Sigma) supplemented with 10 mM EDTA and incubated with either normal human serum (NHS, diluted 1∶10) or Factor H (100 µg/ml, Aventis Behring) for 1 h at 37°C with agitation. Subsequently, the cells were washed four times with EDTA-GVB^2+^ and bound proteins were eluted with SDS buffer (60 mM Tris-HCl, pH 6.8, 2% SDS, 25% glycerine) for 5 min at 98°C. Wash and elute fractions were separated by SDS-PAGE, transferred to a membrane and analyzed by Western blotting using a polyclonal goat Factor H antiserum (Merck) and horseradish peroxidase (hrp) coupled rabbit anti goat antiserum (DAKO) for detection.

### Cloning, expression and purification of recombinant Sbi constructs

Recombinant fragments of the N-terminal region of Sbi (adjacent to the poly-proline region) were engineered, expressed and purified as described previously by (Burman et al. JBC in press). The following Sbi constructs were used in this study: Sbi-E (amino acids 28–266) containing IgG-binding domains I and II and C3 interacting domains III and IV; Sbi-I (amino acids 42–94); Sbi-III-IV (amino acids 150–266) and Sbi-IV (amino acids 197–266).

### Expression of Factor H deletion mutants

The Factor H deletion mutants SCRs 1–7, SCRs 8–11, SCRs 11–15, SCRs 15–18 and SCRs 19–20 were expressed in insect cells infected with recombinant baculovirus as described earlier [39]. Briefly, *Spodoptera frugiperda* cells (Sf9) were grown at 28°C in monolayer cultures in protein-free expression medium for insect cells (BioWhittaker). Adherent Sf9 cells were infected with recombinant virus using a multiplicity of infection of five. The culture supernatant was harvested after 9 days and recombinant Factor H constructs were purified by affinity chromatography using Ni-NTA-Agarose (Qiagen).

### Antibody binding to Sbi-fragments

The complete extra cellular region, Sbi-E, and the extra cellular deletion mutants Sbi-I, Sbi-III/IV, Sbi-IV, BSA (2 µg/ml each) and Factor H (1 µg/ml) were immobilized onto a microtiter plate for 2 h at room temperature. Unspecific binding sites were blocked with 0.2% gelatine in DPBS (Lonza) over night at 4°C. After extensive washing with PBSI (3.3 mM NaH_2_PO_4_×H_2_O, 6.7 mM Na_2_HPO_4_, 145 mM NaCl, pH 7.2) supplemented with 0.05% Tween 20 a polyclonal rabbit SCR1–4 antiserum and the two mABs B22 and C18 (all specific for Factor H) were added for 2 h at room temperature. Protein-antibody complexes were detected using secondary horseradish peroxidase (HRP)-coupled antiserum (e.g. rabbit anti goat-hrp (DAKO) rabbit anti mouse-hrp (DAKO)) Respectively. All antibodies and antisera were used at 1∶1000 dilutions. The reaction was developed with 1,2-phenylenediamine dihydrochloride (OPD, Dako) and the absorbency was measured at 490 nm.

### Protein binding assay - CEWA

A combined ELISA and Western blot approach (CEWA) was used to assay Factor H binding to Sbi-E and the deletion constructs Sbi-I, Sbi-III/IV and Sbi-IV [36]. The proteins (10 µg/ml) were immobilized onto a microtiter plate over night at 4°C. After blocking with 0.2% gelatine in DPBS (Lonza) for 6 h at 4°C, NHS (diluted 1∶10), Factor H (5 µg/ml), a combination of Factor H (5 µg/ml) and C3b (10 µg/ml, Merck), or Factor H (5 µg/ml) and C3d (2,6 µg/ml, Merck) were added. For the C3-CEWA a mixture of Factor H (5 µg/ml) and C3b or C3, iC3b, C3d (each 10 µg/ml, Merck), C3c, C3a (each 10 µg/ml, Comptech) were added. Samples were incubated over night at 4°C. After extensive washing protein complexes were removed with SDS buffer, separated by SDS-PAGE and analyzed by Western blotting using a polyclonal anti C3 antibody (Calbiochem) and anti-goat – hrp (DAKO) was used for the detection of C3 and its degradation products. For Factor H detection the mAB C18, which is specific for SCR 20 of Factor H and rabbit anti mouse-hrp (DAKO) as secondary antibody was used. As positive controls the borrelial Factor H binding protein CRASP-1, and also the Factor H/FHR1 binding protein CRASP-5 (kindly provided by Dr. Peter Kraiczy (University of Frankfurt a. M.) and by Prof. Dr. Reinhard Wallich (University of Heidelberg)) and as negative control BSA were used.

### Enzyme-linked immunosorband assay (ELISA)

The Factor H deletion constructs SCRs 1–7, SCRs 8–11, SCRs 11–15, SCRs 15–18, SCRs 19–20 and SCRs 15–20 were immobilized equimolar onto a microtiter plate over night at 4°C. After blocking with Blocking Buffer I (AppliChem) for 2 h at 37°C, a combination of C3b (5 µg/ml) and the Sbi deletion mutants Sbi-E, Sbi-I and Sbi-III/IV used at equimolar amounts were added and incubated for 1 h at room temperature. After excessive washing bound Sbi deletion mutants were detected with polyclonal Sbi antiserum (1∶1000) and a secondary horseradish peroxidase-coupled anti rabbit antiserum (1∶1000, DAKO). To analyze the complex formation, Sbi-III/IV (10 µg/ml) was coated and a combination of Factor H (15 µg/ml) and C3, C3b, C3d, C3c (Calbiochem) or C3a (15 µg/ml; Comptech) was added. The complex was detected by polyclonal goat anti Factor H (1∶1000) and rabbit anti goat-hrp (1∶1000, Dako).The reaction was developed with 1,2-phenylenediamine dihydrochloride (OPD, Dako) and the absorbency was measured at 490 nm.

### Inhibition of protein binding with an antiserum

Sbi-E and the extra cellular deletion mutants Sbi-I, Sbi-III/IV, Sbi-IV or CRASP-1, and BSA (10 µg/ml) were immobilized and unspecific binding sites were blocked as described. Factor H (5 µg/ml), polyclonal Factor H antiserum (diluted 1∶100) and C3b (10 µg/ml) were preincubated for 2 h at 4°C. Subsequently the mixture was added to the immobilized proteins and incubated over night at 4°C. After extensive washing protein complexes were removed from the well with SDS buffer, separated by SDS-PAGE and analyzed by Western blotting with the polyclonal rabbit SCR1–4 antiserum and swine anti rabbit-hrp (DAKO) as secondary antibody.

### Complement cofactor assay

For determining the regulatory activity of Sbi-bound Factor H, the regulator (3 µg/ml) together with C3b (6 µg/ml) or C3b (6 µg/ml) alone were added to immobilized Sbi-E, Sbi-I, Sbi-III/IV, Sbi-IV, CRASP-1, or BSA (10 µg/ml) incubated over night at 4°C. After extensive washing Factor I (0.8 µg) was added and the mixture was incubated for 30 min at 37°C. C3b conversion to inactive C3b (iC3b) was detected after separating the protein solution by SDS-PAGE with Western blot analysis using a polyclonal goat C3 antiserum (1∶1000, Merck) and rabbit anti goat-hrp (DAKO) as secondary antibody.

### SDS-PAGE and Western blot analysis

Samples were separated by SDS-PAGE using 10% and 12% gels. After the transfer of the proteins onto nitrocellulose membranes by semi-dry blotting [40], the membranes were blocked with 5% (w/v) dried milk in PBSI for 30 min at room temperature and incubated with the indicated primary antibodies over night at 4°C. Antibodies were diluted in 2.5% (w/v) dried milk in PBSI. The proteins were detected by ECL using appropriate secondary antisera that was coupled with horseradish peroxidase.

### Surface plasmon resonance studies

Protein-protein interactions were analyzed by the surface plasmon resonance technique using a Biacore 3000 instrument (Biacore AB) as described [41]. Briefly, the staphylococcal proteins Sbi-E, Sbi-I, Sbi-III/IV or human C3d were coupled to the surface of the flow cells of the sensor chip via a standard amine-coupling procedure (carboxylated dextran chip CM5, Biacore AB) until about 2000 resonance units were reached. A control cell was prepared under identical conditions that lacked a protein. Sbi-E, Factor H, C3, C3b or C3d were diluted in DPBS (Lonza), adjusted to equal molarities and injected with a flow rate of 5 µl/min at 25°C. Alternatively, Ni^2+^ and Sbi-E was loaded to a NTA-chip, and C3d followed by Factor H were injected at equimolar amounts. Each interaction was analyzed at least three times.

### Hemolytic assays

In order to analyze the complement regulatory effect of Sbi, hemolytic assays were performed using rabbit erythrocytes (rE, Rockland). Rabbit erythrocytes represent activator surfaces for human serum and lyse due to MAC formation. Thus the complement activity correlates directly with the erythrocyte lysis as monitored by the increase in absorbance. Following preincubation of NHS with Sbi-E or Sbi-III/IV for 30 min at 37°C, 5×10^6^ rE were added (150 µl total volume) and further incubated for 30 min at 37°C. After centrifugation (2 min, 5000 rpm) the absorbency of the supernatant was measured at 414 nm. NHS, Sbi-E and Sbi-III/IV were used at the indicated concentrations. Samples were diluted in HEPES buffer (20 mM HEPES, 144 mM NaCl, 7 mM MgCl_2_, 10 mM EGTA, 1% BSA, pH 7.4). The effect of Sbi on different animal sera (Innovative Research) was assayed using 30% animal serum and 2 µg (13 µg/ml) Sbi-E.

In order to analyze and distinguish between the alternative and the classical/lectin pathway complement activation was pursued in different buffers. Alternative pathway activity was measured in EGTA-HEPES buffer. Activation of all three pathways was assayed in Ca^2+^-HEPES buffer (20 mM HEPES, 144 mM NaCl, 5 mM CaCl_2_, 2,5 mM MgCl_2_, pH 7.4). The effect of the classical and the lectin pathway was assayed in Factor B deficient serum (Complement Technology Inc.) and the Ca^2+^-HEPES buffer. All three approaches (AP, AP+CP/LP and CP/LP) were analyzed in the presence of none, 0.3 µg (2 µg/ml) and 1.0 µg (6,7 µg/ml) Sbi-E.

### *S.aureus* survival assay

Bacteria *S. aureus* strain H591 (6×10^4^) were incubated in 40% NHS supplemented with HEPES EGTA in presence or absence of Sbi-E for 15 min at 37°C. Samples were added to 8×10^5^ PMA primed THP-1 macrophages in antibiotic free RPMI-1640 resulting in a final Sbi-E concentration of 2 µg/ml. THP-1 cells incubated without *S. aureus* were used as negative control. After shaking 20 µl sample were plated hourly. Plates were incubated overnight and colonies were counted.

### Accession codes

National Centre for Biotechnology Information (www.ncbi.nlm.nih.gov): Homo sapiens complement factor H (CFH), gi|62739185|ref|NM_000186.2|[62739185]; Homo sapiens complement factor H-related 1 (CFHR1), NM_002113.2 GI:118442838; Homo sapiens complement component 3 (C3), NM_000064.2 GI:115298677; immunoglobulin G-binding protein Sbi [Staphylococcus aureus subsp. aureus str. Newman], YP_001333351.1 GI:151222529.

## Supporting Information

Figure S1 Characterization of H591. *S. aureus* H591 were grown overnight on LB-Agar. Individual colonies were picked and lysed in 50 μl dH2O for 10 min at 96 °C. A standard PCR was performed using Sbi specific Primers (Sbi-fw GCGAGTGAAAACACGCAACA, Sbi-rev CGCCACTTTCTTTTCAGCAT). Samples were analyzed on a 1% agarose-gel. To analyze the existence and location of the Sbi protein for *S. aureus* H591, a 20 ml overnight culture was separated. The supernatant was concentrated 10-fold using the Centricon-Plus-20 (Millipore) concentrators. The resulting cell-pellet was disintegrated by bead beating (Mini-BeadBeater-1, BioSpec). The extract was centrifuged at 13200 rpm for 10 min. The supernatant containing soluble proteins was defined as “cytoplasm” fraction. The residual pellet was washed with 1x PBS, separated from the glass beads and defined as “cell-wall” fraction. Samples were separated SDS-Page followed by Western blotting. Sbi was detected using specific anti Sbi F(ab’)2 Fragments generated with the F(ab’)2 Preparation Kit (Pierce). Anti-rabbit F(ab’)2 -hrp (Santa Cruz Biotechnology) was used as secondary antibody. Protein A (Sigma) was used as control. Protein A (1 μg) and Sbi-E (1 μg) were separated on SDS page in triplicate and Western blotted. The blot was cut in three parts and proteins were detected using following antibody combinations: anti-Sbi F(ab’)2 and goat anti-rabbit F(ab’)2 - hrp, rabbit anti-goat - hrp, rabbit Sbi antiserum and goat anti-rabbit F(ab’)2 - hrp. (A) *S. aureus* strain H591 expresse Sbi. Sbi was detected in the cytoplasmic and cell-wall fraction and a truncated form in the concentrated supernatant. Sbi was detected using polyclonal rabbit anti-Sbi F(ab’)2 and goat anti-rabbit F(ab’)2 - hrp as secondary antibody. (B) FACS analysis confirmed the presence of Sbi on surface of *S. aureus*. Anti-Sbi F(ab’)2 and Alexa488 coupled anti-rabbit F(ab’)2 was used for detection. An unspecific Alexa488 coupled rabbit F(ab’)2 and the secondary F(ab’)2 was used as negative controls (C) *S. aureus* H591 carries the Sbi encoding gene. The sbi-gene was amplified using specific primers. (D) The *sbi*-gene of four separate colonies was sequenced and aligned to the sbi-gene of *S. aureus* Newman strain. Six sequence variations were detected and one affects the protein-sequence V169I. (E) Anti-Sbi F(ab’)2 shows no crossreactivity with Protein A (SpA). SpA and Sbi-E was separated on SDS page in triplicate and analyzed by Western blotting. Proteins were detected using following antibody combinations: anti-Sbi F(ab’)2 and a secondary goat anti-rabbit F(ab’)2- hrp (Lane 1 and 2), rabbit anti-goat - hrp (Lane 3 and 4), Sbi antiserum and the secondary anti-rabbit F(ab’)2 - hrp (Lane 5 and 6).
(0.26 MB PDF)

Figure S2 1:1 model (langmuir). (A) Concentration-dependent interaction of the C3d to immobilized Sbi-E was recorded in real time by surface plasmon resonance. A kinetic model shows that this profile does not fit a 1:1 langmuir interaction. Solid lanes representing the calculated data do not match the experimental data. (B) The experimental data fit a theoretical 2:1 interaction much better, as experimental and fitted data show a good match and rather low deviation. The residual blots (C,D) show the difference between the experimental and the fitted data for each curve. This presentation reveals systematic deviations between the experimental and the calculated data. (C) The curves for a 1:1 interaction show a large variation. (D) The 2:1 interaction model show a much better fit between the experimental and fitted data. The deviation is very low. For a perfect fit, the scatter in the residual plot is a measure of the noise in the signal. Normally the noise levels are in */- 2 RU.
(0.29 MB PDF)

Figure S3 Immobilization of Sbi I onto the surface of the sensor chip was followed. (A) Upon sequential injection of the protein, a bulk effect is observed. When the probe was washed, an increase in the base line is detected which demonstrates immobilization of the ligand. This step was repeated four times until a level of immobilization of approximately 1,800 RU was detected (D). (B) An unspecific mouse IgG preparation binds to immobilized Sbi-I, thus demonstrating that immobilized Sbi-I is accessible and binds ligands. Similarly, IgG did also bind to the immobilized IgG binding Sbi-E. No binding was observed for the NON IgG binding Sbi-III/IV fragment. This profile represents the unspecific bulk effect. However, when binding of C3d was analysed, the immobilized Sbi-III/IV fragment showed binding (compare Figure 5).
(0.01 MB PDF)
