# Supplementary material for: The Staphylococcus aureus Protein Sbi Acts as a Complement Inhibitor and Forms a Tripartite Complex with Host Complement Factor H and C3b
Source: PLoS Pathog. 2008 Dec 26;4(12):e1000250. doi: 10.1371/journal.ppat.1000250 (PMC2602735; doi:10.1371/journal.ppat.1000250)
Supplement: Figure S1 — Characterization of H591. S. aureus H591 were grown overnight on LB-Agar. Individual colonies were picked and lysed in 50 μl dH2O for 10 min at 96 °C. A standard PCR was performed using Sbi specific Primers (Sbi-fw GCGAGTGAAAACACGCAACA, Sbi-rev CGCCACTTTCTTTTCAGCAT). Samples were analyzed on a 1% agarose-gel. To analyze the existence and location of the Sbi protein for S. aureus H591, a 20 ml overnight culture was separated. The supernatant was concentrated 10-fold using the Centricon-Plus-20 (Millipore) concentrators. The resulting cell-pellet was disintegrated by bead beating (Mini-BeadBeater-1, BioSpec). The extract was centrifuged at 13200 rpm for 10 min. The supernatant containing soluble proteins was defined as “cytoplasm” fraction. The residual pellet was washed with 1x PBS, separated from the glass beads and defined as “cell-wall” fraction. Samples were separated SDS-Page followed by Western blotting. Sbi was detected using specific anti Sbi F(ab’)2 Fragments generated with the F(ab’)2 Preparation Kit (Pierce). Anti-rabbit F(ab’)2 -hrp (Santa Cruz Biotechnology) was used as secondary antibody. Protein A (Sigma) was used as control. Protein A (1 μg) and Sbi-E (1 μg) were separated on SDS page in triplicate and Western blotted. The blot was cut in three parts and proteins were detected using following antibody combinations: anti-Sbi F(ab’)2 and goat anti-rabbit F(ab’)2 - hrp, rabbit anti-goat - hrp, rabbit Sbi antiserum and goat anti-rabbit F(ab’)2 - hrp. (A) S. aureus strain H591 expresse Sbi. Sbi was detected in the cytoplasmic and cell-wall fraction and a truncated form in the concentrated supernatant. Sbi was detected using polyclonal rabbit anti-Sbi F(ab’)2 and goat anti-rabbit F(ab’)2 - hrp as secondary antibody. (B) FACS analysis confirmed the presence of Sbi on surface of S. aureus. Anti-Sbi F(ab’)2 and Alexa488 coupled anti-rabbit F(ab’)2 was used for detection. An unspecific Alexa488 coupled rabbit F(ab’)2 and the secondary F(ab’)2 was used as negati [file ppat.1000250.s001.pdf]

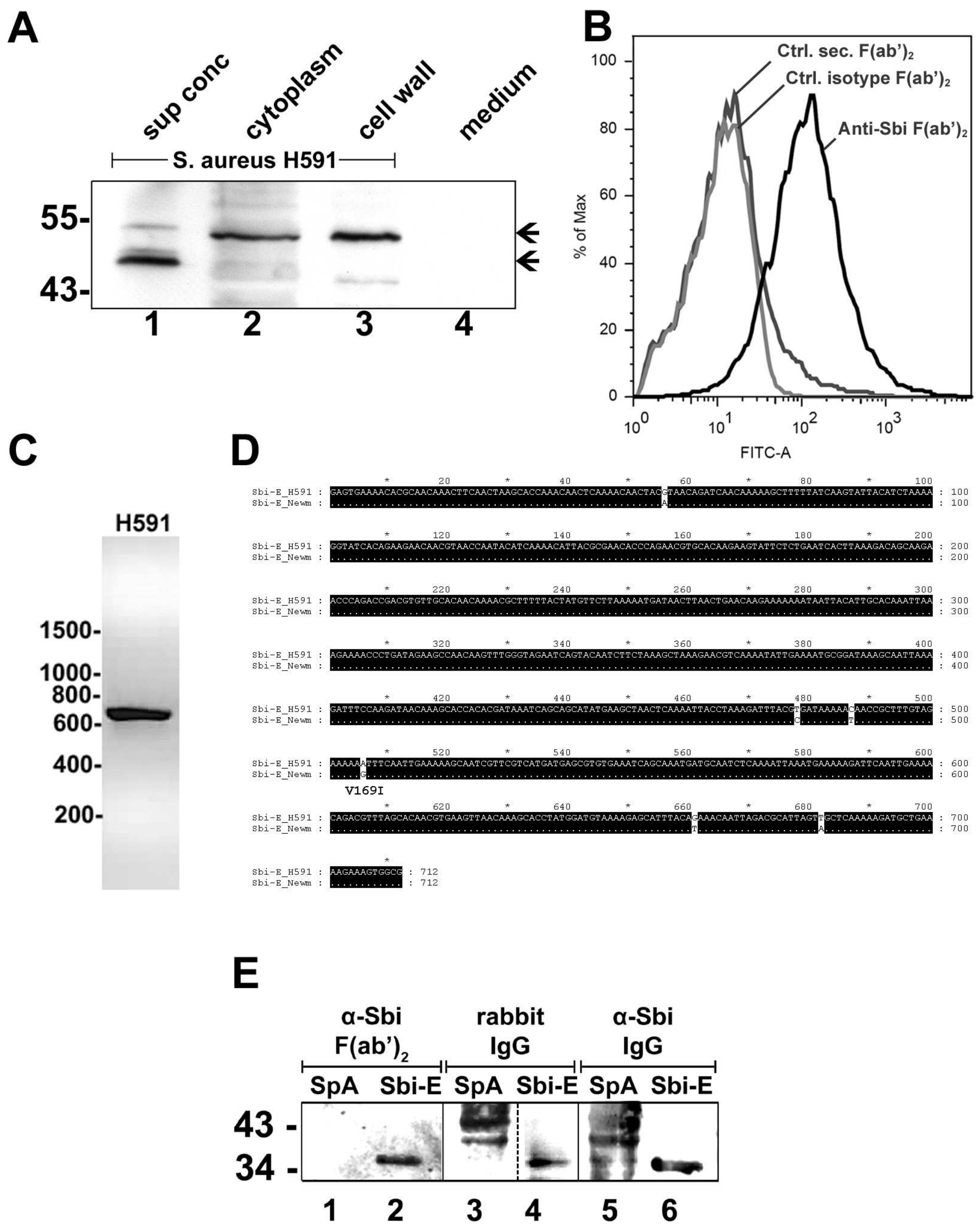
A
sup conc
cytoplasm
cell wall
medium
S. aureus H591
55-
43-
1
2
3
4
B
Ctrl. sec. F(ab')2
Ctrl. isotype F(ab')2
Anti-Sbi F(ab')2
% of Max
FITC-A
C
H591
1500-
1000-
800-
600-
400-
200-
D
Sbi-E_H591
Sbi-E_Newm
V169I
E
α-Sbi F(ab')2
rabbit IgG
α-Sbi IgG
SpA
Sbi-E
43 -
34 -
1
2
3
4
5
6

Supporting information S1

Characterization of H591

S. aureus H591 were grown overnight on LB-Agar. Individual colonies were picked and lysed in 50 µl dH_2O for 10 min at 96 °C. A standard PCR was performed using Sbi specific Primers (Sbi-fw GCGAGTGAAAACACGCAACA, Sbi-rev CGCCACTTTCTTTTCAGCAT). Samples were analyzed on a 1% agarose-gel.

To analyze the existence and location of the Sbi protein for *S. aureus* H591, a 20 ml overnight culture was **separated**. The supernatant was concentrated 10-fold using the Centricon-Plus-20 (Millipore) concentrators. The resulting cell-pellet was disintegrated by bead beating (Mini-BeadBeater-1, BioSpec). The extract was centrifuged at 13200 rpm for 10 min. The supernatant containing soluble proteins was defined as "cytoplasm" fraction. The residual pellet was washed with 1x PBS, separated from the glass beads and defined as "cell-wall" fraction. Samples were separated SDS-Page followed by Western blotting. Sbi was detected using specific anti Sbi F(ab')2 Fragments generated with the $F(ab')_2$ Preparation Kit (Pierce). Anti-rabbit F(ab')2 –hrp (Santa Cruz Biotechnology) was used as secondary antibody. Protein A (Sigma) was used as control.

Protein A (1 µg) and Sbi-E (1 µg) were separated on SDS page in triplicate and Western blotted. The blot was cut in three parts and proteins were detected using following antibody combinations: anti-Sbi F(ab')2 and goat anti-rabbit F(ab')2 – hrp, rabbit anti-goat – hrp, rabbit Sbi antiserum and goat anti-rabbit F(ab')2 – hrp.

Supplementary figure 1: (A) *S. aureus* strain H591 expresse Sbi. Sbi was detected in the cytoplasmic and cell-wall fraction and a truncated form in the concentrated supernatant. Sbi was detected using polyclonal rabbit anti-Sbi F(ab')2 and goat anti-rabbit F(ab')2 – hrp as secondary antibody. (B) FACS analysis confirmed the presence of Sbi on surface of *S. aureus*. Anti-Sbi F(ab')2 and Alexa488 coupled anti-rabbit F(ab')2 was used for detection. An unspecific Alexa488 coupled rabbit F(ab')2 and the secondary F(ab')2 was used as negative controls (C) *S. aureus* H591 carries the Sbi encoding gene. The *sbi*-gene was amplified using specific primers. (D) The *sbi*-gene of four separate colonies was sequenced and aligned to the sbi-gene of *S. aureus* Newman strain. Six sequence variations were detected and one affects the protein-sequence V169I. (E) Anti-Sbi F(ab')2 shows no crossreactivity with Protein A (SpA). SpA and Sbi-E was separated on SDS page in triplicate and analyzed by Western blotting. Proteins were detected using following antibody combinations: anti-Sbi F(ab')2 and a secondary goat anti-rabbit F(ab')2– hrp (Lane 1 and 2), rabbit anti-goat – hrp (Lane 3 and 4), Sbi antiserum and the secondary anti-rabbit F(ab')2 – hrp (Lane 5 and 6).
