# Supplementary figures and images for: The Staphylococcus aureus Protein Sbi Acts as a Complement Inhibitor and Forms a Tripartite Complex with Host Complement Factor H and C3b
Source: PLoS Pathog. 2008 Dec 26;4(12):e1000250. doi: 10.1371/journal.ppat.1000250 (PMC2602735; doi:10.1371/journal.ppat.1000250)

A

1:1 model (langmuir)

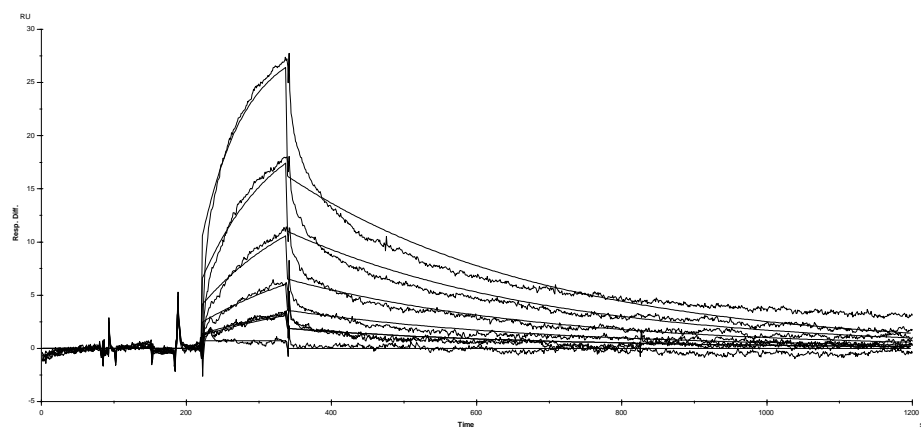

B

2:1 model

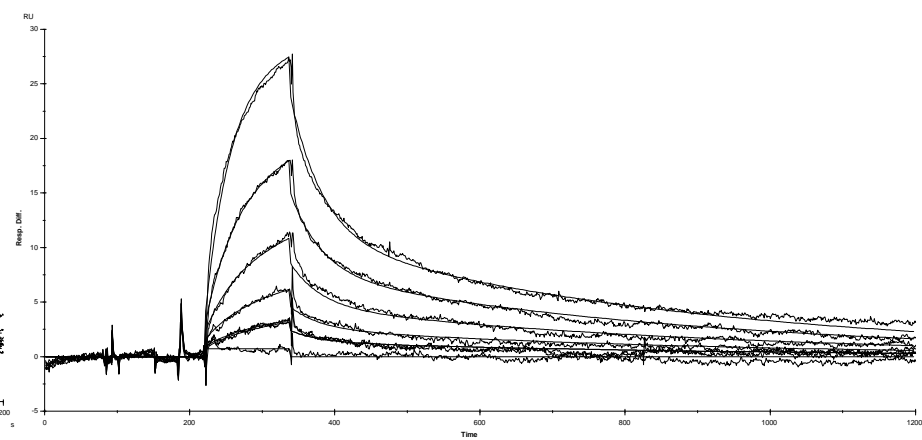

C

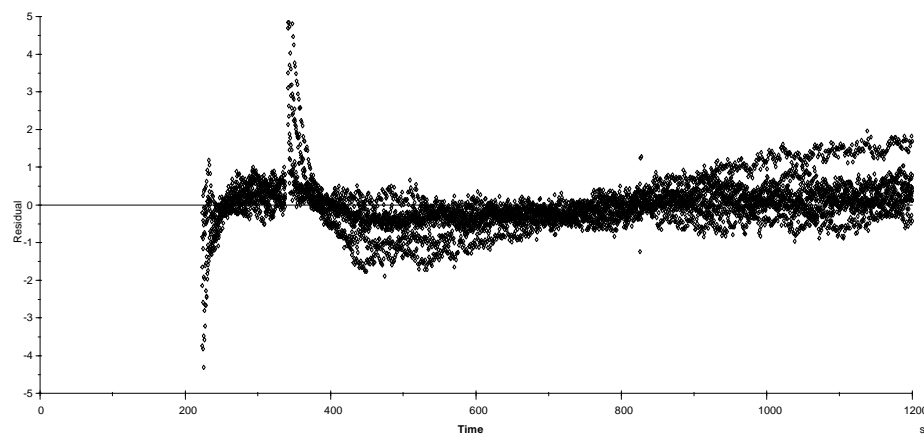

D

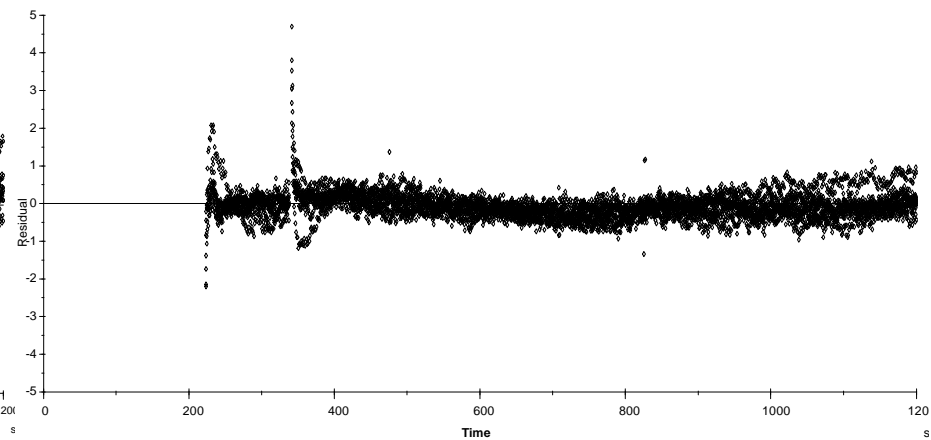

Supplement: Figure S2 — 1:1 model (langmuir). (A) Concentration-dependent interaction of the C3d to immobilized Sbi-E was recorded in real time by surface plasmon resonance. A kinetic model shows that this profile does not fit a 1:1 langmuir interaction. Solid lanes representing the calculated data do not match the experimental data. (B) The experimental data fit a theoretical 2:1 interaction much better, as experimental and fitted data show a good match and rather low deviation. The residual blots (C,D) show the difference between the experimental and the fitted data for each curve. This presentation reveals systematic deviations between the experimental and the calculated data. (C) The curves for a 1:1 interaction show a large variation. (D) The 2:1 interaction model show a much better fit between the experimental and fitted data. The deviation is very low. For a perfect fit, the scatter in the residual plot is a measure of the noise in the signal. Normally the noise levels are in */- 2 RU. (0.29 MB PDF) [file ppat.1000250.s002.pdf]

A

### Immobilisation of Sbi-I

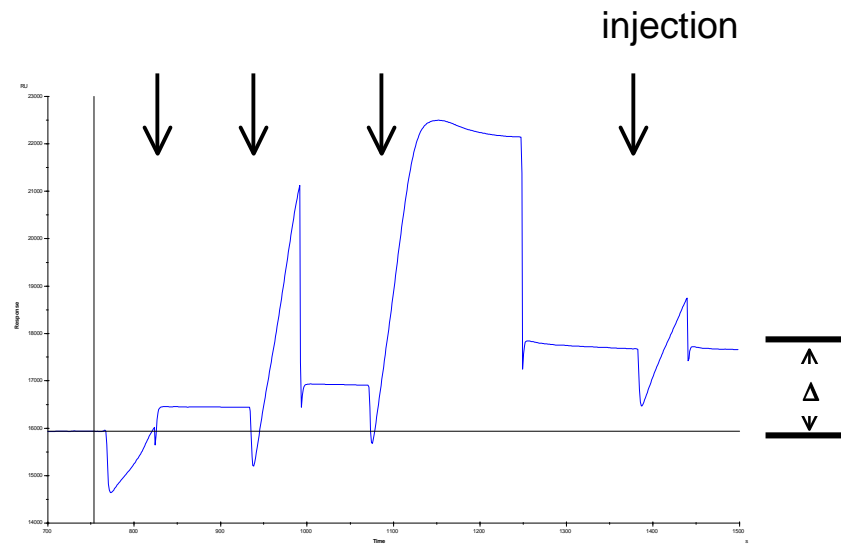

B

### Binding of an unrelated mouse IgG to immobilized Sbi-I

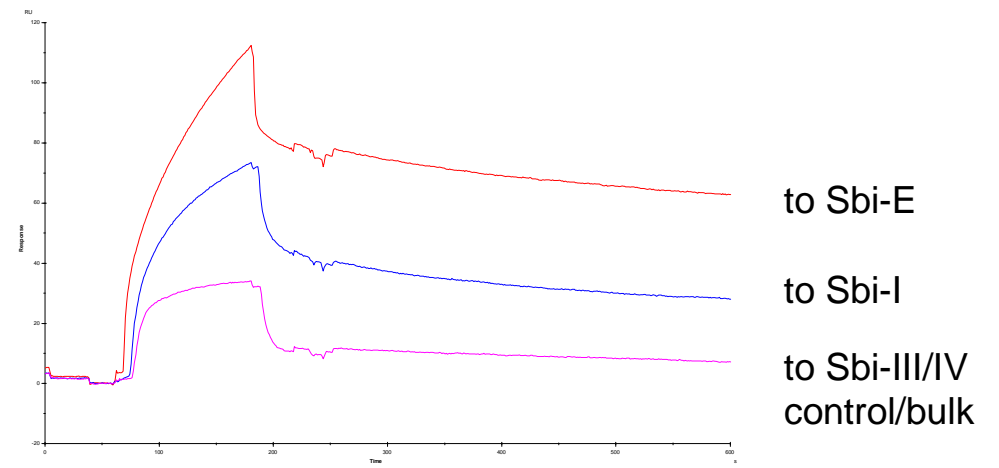

Supplement: Figure S3 — Immobilization of Sbi I onto the surface of the sensor chip was followed. (A) Upon sequential injection of the protein, a bulk effect is observed. When the probe was washed, an increase in the base line is detected which demonstrates immobilization of the ligand. This step was repeated four times until a level of immobilization of approximately 1,800 RU was detected (D). (B) An unspecific mouse IgG preparation binds to immobilized Sbi-I, thus demonstrating that immobilized Sbi-I is accessible and binds ligands. Similarly, IgG did also bind to the immobilized IgG binding Sbi-E. No binding was observed for the NON IgG binding Sbi-III/IV fragment. This profile represents the unspecific bulk effect. However, when binding of C3d was analysed, the immobilized Sbi-III/IV fragment showed binding (compare Figure 5). (0.01 MB PDF) [file ppat.1000250.s003.pdf]
